# PLpro Inhibitors as a Potential Treatment for COVID-19

**DOI:** 10.3390/biomedicines13061417

**Published:** 2025-06-10

**Authors:** Yu Han, Feng Xu

**Affiliations:** Department of Infectious Diseases, The Second Affiliated Hospital of Zhejiang University School of Medicine, Hangzhou 310009, China

The advent of severe acute respiratory syndrome coronavirus 2 (SARS-CoV-2) and the subsequent coronavirus disease 2019 (COVID-19) pandemic have posed a serious threat to human health and society [1]. SARS-CoV-2 is a coronavirus that causes respiratory tract infections that can lead to severe outcomes, including mortality [2]. Although vaccines greatly alleviated the harm caused by the pandemic, the swift spread and constant evolution of new variants such as Omicron and Delta reduced vaccine efficacy and continue to threaten global public health. Therefore, exploring new targets and developing new drugs to overcome SARS-CoV-2 drug resistance is currently a top priority in drug research and development related to COVID-19.

Coronavirus replication relies on the coordinated actions of essential enzymes, including the main protease (3CLpro/Mpro), RNA-dependent RNA polymerase (RdRp), and papain-like protease (PLpro) [3]. These enzymes are the targets of anti-coronavirus drugs that have been a major focus of research in recent years [4]. Thus far, the FDA has approved three antiviral drugs, including two inhibitors of RdRp (remdesivir and molnupiravir) and one inhibitor of Mpro (Nirmatrevir) [5]. Notably, viruses with mutations conferring resistance to remdesivir or nirmatrelvir have been identified in both cell culture-based serial passage experiments and treated COVID-19 patients. [6,7]. In the mutant of SARS-CoV-2, PLpro is highly conserved, which makes it a much-concerned antiviral drug target [8]. The development of PLpro inhibitors will open the doors for testing new strategies, tools and drugs against SARS-CoV-2 infection.

This Editorial briefly reviews some recent studies on the development of PLP crystal structures and PLP inhibitors. We summarize a variety of research contributions that look to the future prospects and challenges associated with PLP inhibitors.

## 1. Role of PLpro in SARS-CoV-2 Infection

As an essential catalytic component for SARS-CoV-2 replication, PLpro cleaves viral polyproteins to generate functional replicase complexes while facilitating viral dissemination [9]. Furthermore, PLpro modulates host-pathogen interactions through post-translational modification of cleaved host proteins, thereby enacting an immune evasion strategy against antiviral defenses. This protease specifically hydrolyzes the ISG15, catalyzing deISGylation [9]. ISG15 is a type I interferon (IFN)-induced gene that mediates ISGylation, a covalent conjugation process modifying at least 150 proteins to regulate signaling pathways including NF-κB, JNK, and IRF-3 signaling [10]. ISG15 also exists in free form and functions intracellularly or extracellularly [11]. The post-transcriptional modification of ISG15 is often targeted by viruses, aiming to interfere with the host immune response by deISGylation to counteract the effects [12]. Notably, SARS-CoV-2 PLpro dysregulates the equilibrium between free and conjugated ISG15, correlating with diminished pro-inflammatory phenotypes and impaired antigen presentation in macrophages [13]. Thus, targeting PLpro offers a dual therapeutic advantage: inhibiting viral replication and restoring host immune homeostasis.

## 2. Advances in the Crystal Structure of SARS-CoV-2 PLpro

Recently, with the rapid development of structural biology and crystallography technology, scientists have conducted in-depth research on the crystal structure of PLpro. In 2020, many research teams published results on the crystal structure of PLpro in prestigious journals, which provided valuable structural information for the development of COVID-19 drugs. An article entitled “Crystal structure of SARS-CoV-2 papain-like protease” was published in *Acta Pharmaceutica Sinica B* [14]. It was the first time that a research team had analyzed the ligand-free structure of SARS-CoV-2 PLpro and compared its high-resolution three-dimensional structure with that of GRL0617, a highly effective PLpro inhibitor [14]. At the same time, two international research teams published research papers on PLpro in *Nature* [9] and *The EMBO Journal* [15], respectively. Shin et al. resolved the crystal structure of SARS-CoV-2 PLpro in complex with ISG15. Both research teams resolved the crystal structure of the complex between PLpro and the native substrates of the host protein but did not obtain the crystal structure of the GRL0617 or its derivatives. Nevertheless, they proposed a GRL0617 mechanism of action through molecular simulation, which complemented the findings of the Chinese Academy of Sciences team and jointly contributed to drug development.

## 3. Progress in the Research and Development of PLpro Inhibitors Against SARS-CoV-2

Multiple candidate inhibitors targeting PLpro have emerged in recent research. In vitro studies demonstrate that the generic compound 6-thioguanine (6-TG) suppresses SARS-CoV-2 replication in Vero-E6 cell cultures [16]. Similarly, disulfiram, an FDA-approved therapeutic, exhibits inhibitory activity against SARS-CoV and MERS PLpro isoforms [17]. However, the clinical efficacy of these two approved drugs is limited.

GRL0617, a non-covalent inhibitor, achieves an IC50 of 2.4 μM against SARS-CoV-2 PLpro [18]. However, GRL0617 lacks sufficient stability and efficacy to further develop antiviral drugs. It is sufficiently effective as an antiviral agent, and its eutectic structure provides a structural template for designing more effective SARS-CoV-2 PLpro inhibitors. Significantly, the method utilizes GRL0617 as a template, employing an ~7 Å linker to reach the active site cysteine, thereby generating covalent inhibitors exhibiting double-digit nanomolar potency [19]. Unfortunately, the most potent inhibitors in this series were reported to be unsuitable for the research in animal models.

The article entitled “Design of a SARS-CoV-2 Papain-like Protease Inhibitor with Antiviral Efficacy in a Mouse Model” presents promising results, demonstrating antiviral activity against SARS-CoV-2 variants, including those resistant to nirmatrelvir [20]. The use of structural biology to design a compound targeting both the BL2 groove and the Val70Ub binding pocket is a significant contribution to the field. The data presented, particularly the antiviral efficacy in a mouse model, support the potential of this compound for future clinical development. The dual targeting of the BL2 groove and Val70Ub sites is innovative and is expected to overcome the challenge of viral resistance. Although the molecular basis of inhibition is outlined relatively clearly in the present paper, the elucidation of the molecular dynamics and interactions between Jun12682 and PLpro may facilitate an in-depth study of its inhibition mechanism. The role of the Val70Ub binding site in drug resistance prevention can be further explored. In the COVID-19-infected mouse model, the oral administration of Jun12682 not only significantly improved mouse survival rate but also significantly reduced mouse lung virus titer and tissue damage, as well as the expression of a variety of inflammatory factors, demonstrating excellent in vivo activity. In addition, the inhibitory activity of Jun12682 against drug-resistant virus strains highlighted the therapeutic potential of the compound. However, its short half-life and the lack of toxicology or long-term safety data in animal models suggest that clinical transformation remains a challenge. Therefore, Jun12682 will benefit from additional details regarding pharmacokinetics, long-term safety, and the potential for combination therapy. Solving these problems will provide a clearer roadmap for the clinical application of this compound. Further design optimization, improvement in pharmacokinetics, long-term safety evaluation, and other issues will facilitate the clinical application of this compound.

An article entitled “Discovery of SARS-CoV-2 papain-like protease (PLpro) inhibitors with efficacy in a murine infection model” published in *Science Advances* represents an important contribution to the exploration of new antiviral therapies for COVID-19. The authors describe the identification of novel inhibitors targeting the SARS-CoV-2 PLpro enzyme [21]. The research provided promising data, proved the potential of these inhibitors against SARS-CoV-2, and proposed a mouse model to further verify its therapeutic effect [21].

Another research team from Guangzhou National Laboratory published an article online in *Nature Communications*, entitled “Discovery of orally bioavailable SARS-CoV-2 papain-like protease inhibitor as a potential treatment for COVID-19”. The study discovered a novel PLpro inhibitor GZNL-P36 with high oral bioavailability, which has shown better antiviral activity than the reported PLpro inhibitor in mouse animal models and may be a potential treatment for COVID-19 infection [22]. Similarly, GZNL-P36 can inhibit the PLpro activity of different coronaviruses, which has the potential to be a broad-spectrum anti-coronavirus [22]. While the manuscript provides convincing data on the efficacy of GZNL-P36, further discussion of the detailed mechanisms of PLpro inhibition will enhance our understanding of this topic. Whether off-target effects that could affect its clinical application require further study remains to be determined.

## 4. Prospects and Challenges of PLpro Inhibitors in Drug Research and Development

Based on the protein structure analysis of PLpro, it has two potential small molecule inhibitor binding pockets: the catalytic domain and the BL2 domain.The catalytic domain of PLpro is closed under normal conditions and opened when the substrate approaches, whereas the catalytic domain of MPro is always open. Therefore, small molecules have difficulty binding to the PLpro catalytic domain, and the current enzyme activity inhibition of small-molecule inhibitors still needs improvement. The BL2 domain of PLpro is highly dynamic, and the binding force is not high due to the loss of free energy when binding with small molecular inhibitors. Compared with MPro and RdRp inhibitors, PLpro inhibitors are a new track full of challenges and opportunities. Furthermore, the variability and drug resistance of the SARS-CoV-2 virus are also significant issues in drug research and development. In addition, it is also necessary to study the safety, effectiveness and clinical applicability of drugs. However, PLpro still has huge potential as a new growth point of COVID-19 drug research and development, despite many challenges. With the continuous development of structural biology, crystallography, medicinal chemistry, and clinical medicine, it is believed that scientists will be able to develop more effective, safe and specific inhibitors against SARS-CoV-2 PLpro and make important contributions to fight against the COVID-19 epidemic.
